# The association between Type D personality and the metabolic syndrome: a cross-sectional study in a University-based outpatient lipid clinic

**DOI:** 10.1186/1756-0500-4-105

**Published:** 2011-04-05

**Authors:** Dimitrios Tziallas, Michael S Kostapanos, Petros Skapinakis, Haralampos J Milionis, Thanos Athanasiou, Moses S Elisaf, Venetsanos Mavreas

**Affiliations:** 1 Department of Psychiatry, University of Ioannina School of Medicine, Ioannina, Greece; 2 Department of Internal Medicine, University of Ioannina School of Medicine, Ioannina, Greece; 3 Division of Surgery, Oncology, Reproductive Biology and Anaesthetics, Faculty of Medicine, Imperial College, London, UK

## Abstract

**Background:**

Type D personality has been associated in the past with increased cardiovascular mortality among patients with established coronary heart disease. Very few studies have investigated the association of type D personality with traditional cardiovascular risk factors. In this study, we assessed the association between type D personality and the metabolic syndrome.

**Findings:**

New consecutive patients referred to an outpatient lipid clinic for evaluation of possible metabolic syndrome were eligible for inclusion in the study. The metabolic syndrome was defined according to the International Diabetes Federation (IDF) diagnostic criteria. Type D personality was assessed with the DS-14 scale. Multivariate regression techniques were used to investigate the association between personality and metabolic syndromes adjusting for a number of medical and psychiatric confounders. Three hundred and fifty-nine persons were screened of whom 206 met the diagnostic criteria for the metabolic syndrome ("cases") and 153 did not ("control group"). The prevalence of type D personality was significantly higher in the cases as compared to the control group (44% versus 15% respectively, p < 0.001). In multivariate logistic regression analysis the presence of Type D personality was significantly associated with metabolic syndrome independently of other clinical factors, anxiety and depressive symptoms (odds ratio 3.47; 95% Confidence Interval: 1.90 - 6.33).

**Conclusions:**

Type D personality was independently associated with the metabolic syndrome in this cross-sectional study. The potential implications of this finding, especially from a clinical or preventive perspective, should be examined in future research.

## Introduction

Over the recent years, there is a growing interest of the impact of various psychosocial factors in cardiovascular morbidity and mortality [1]. Several prospective studies suggested that common mental disorders, such as depression and anxiety, may be associated with an increased risk of cardiovascular events in either healthy persons or patients with coronary heart disease (CHD) [1]. Similarly psychosocial factors, such as enhanced 'job strain' as well as the lack of social support, have been proposed as risk factors for CHD. In this context, it is also noted that specific patterns of personality characteristics have been found to independently predict CHD morbidity and mortality [1].

Type D personality is a relatively new personality construct which is characterized by two global personality traits: negative affectivity and social inhibition [2]. Persons exhibiting this type of personality are inclined to experience negative emotions which are further associated with the tendency to inhibit these emotions by avoiding social contacts [2]. These personality traits have been found to be relatively stable over time and lead to chronic emotional stress [3,4]. They are distinguished from depression or anxiety which is more episodic in nature and tends to change over time [3]. Prospective studies have suggested a potential association of type D personality with poor prognosis and increased mortality rates in patients with established CHD [2,3,5].

Evidence of underlying mechanisms to clarify the relationship between type D personality and the pathogenesis of CHD is just beginning to emerge[1]. Some studies suggested that type D personality may be associated with increased inflammation and enhanced cortisol secretion in response to stress, and these may increase the risk for cardiovascular morbidity[6-8]. Furthermore, individuals with type D personality may also suffer from co-morbid depressive and/or anxiety disorders that may account for worse prognosis in patients with CHD [9].

The metabolic syndrome (MetS) comprises the clustering of traditional cardiovascular risk factors, including obesity, hypertension, dyslipidemia and hyperglycemia [10]. Longitudinal studies have shown that patients with the metabolic syndrome may exhibit greater cardiovascular morbidity and mortality as compared to persons who do not fulfill the diagnostic criteria of the metabolic syndrome [10]. Since MetS can increase the risk of subsequent CHD [10] it would be important to investigate whether the type D personality is also associated with such preliminary stages of disease as the possibility of primary prevention may be more feasible at that time. In a recent community study in the Netherlands [11] an association was reported between type D personality and the metabolic syndrome but the latter was assessed using a self-report questionnaire. In addition type D personality has been associated with some components of the MetS (such as hypertension in women and hypercholesterolemia in men) in a general population sample from Germany [12].

The main aim of the present study was therefore to investigate whether the prevalence of type D personality was higher in patients with the MetS as compared to a control group of persons not fulfilling the criteria for MetS. A variable that could potentially confound this association is the presence of episodic distress (mainly in the form of depressive and anxiety symptoms) since this is known to be associated with both type-D personality and the risk for CHD [13,3]. Therefore our secondary aim was to test the hypothesis that the association between type-D and the MetS would be independent of the presence of anxiety or depressive symptoms.

## Methods

### Study population

This cross-sectional study was conducted in Ioannina, a major university city of the Northwestern part of Greece. New consecutive patients with a presumptive diagnosis of MetS referred to the Outpatient Lipid Clinic of the University Hospital of Ioannina (the referral centre for the broader region of Epirus with 300,000 inhabitants) from April 2007 to January 2009 were eligible for inclusion to the study. Participants were excluded if they had significant medical or psychiatric comorbidities. These included coronary heart disease, types 1 or 2 diabetes, thyroid disorders, severe hepatic or renal diseases. All included persons gave a written informed consent. The study protocol was approved by the Ethics Committee of the University Hospital of Ioannina.

### Clinical evaluation

According to the study protocol, at the first visit of each subject in the Outpatient Lipid Clinic, the following parameters were evaluated: i) anthropometric indices [BMI, waist circumference], ii) smoking habits, iii) blood pressure readings, iv) routine laboratory measurements including fasting blood glucose and serum lipid profile.

Waist circumference was measured by placing a tape in a horizontal plane around the abdomen between the lower rib and iliac crest. The tape should have been snug without compressing the skin and parallel to the floor. Measurements were taken at the end of a normal expiration by the same investigator. Blood pressure was measured in triplicate, following ten minutes rest, at each visit. Measurements were carried out by trained physicians in the sitting position using a standard validated automated sphygmomanometer. The first reading was discarded and the mean of the second and third readings was used for the analysis.

Participants were asked a series of questions about their smoking habits. They were first asked a question about lifetime smoking and then about their smoking status during the past month. Persons who smoked during the past month were also asked the average number of cigarettes smoked per day (grouped in less than 10, 10-20, more than 20 per day). For our analysis, all participants who admitted having smoked during the past month were considered current smokers and the remaining non-smokers.

Height and body weight were measured with participants standing without shoes and heavy outer garments. Body mass index (BMI) was calculated as weight divided by height squared (kg/m^2^).

### Laboratory investigations

All laboratory measurements were performed at the Biochemistry Laboratory of the University Hospital of Ioannina, by standardized methods using an Olympus AU600 clinical chemistry analyzer (Olympus Diagnostica, Hamburg, Germany), as described previously [14]. Blood samples were obtained following a 14-hour overnight fast. The concentrations of total cholesterol and TG were determined enzymatically on the Olympus AU 600 clinical chemistry analyzer. HDL-cholesterol was determined by a direct assay[14]. Low-density lipoprotein (LDL)-cholesterol was calculated using the Friedewald formula. Blood glucose was measured by the hexokinase method [14].

### Diagnosis of Metabolic Syndrome

Metabolic syndrome was defined according to the International Diabetes Federation (IDF) definition for European populations using the following criteria:

- presence of central obesity, as defined either by waist circumference ≥ 94 cm in men or ≥ 80 cm in women or by body mass index (BMI) > 30 mg/m^2^, plus two of the following criteria: i) triglyceride (TG) levels ≥ 150 mg/dL, or specific treatment for this lipid abnormality ii) high-density-lipoprotein (HDL)-cholesterol levels < 40 mg/dL in men and < 50 mg/dL in women, or specific treatment for this lipid abnormality iii) fasting plasma glucose ≥ 100 mg/dL (≥ 5.6 mmol/L) or previously diagnosed type 2 diabetes, and iv) systolic blood pressure ≥ 130 mmHg or diastolic blood pressure ≥85 mmHg or treatment for previously diagnosed hypertension. The diagnosis of hyperglycemia and dyslipidemia was based on the average of two measurements taken at least two weeks apart. If there was a 15% or greater difference between blood glucose or lipid measurements a re-evaluation visit was scheduled and the mean value of the first visit and re-evaluation visit measurements was used for the determination.

According to the above mentioned definition, our population was divided into those persons who fulfilled the diagnostic criteria for the MetS (MetS group; cases) and those who did not fulfill the criteria for the diagnosis of the MetS (control group; controls). A total of 359 persons (206 individuals who fulfilled the diagnostic criteria for the MetS [cases] and 153 individuals who did not meet the criteria for the diagnosis of the MetS [controls]) were included in the present study.

### Assessment of type D personality

All participants completed the Type D Scale-14 (DS14) which is designed to assess the Type D personality [4]. Type D refers to the tendency to simultaneously (i) experience negative emotions and (ii) inhibit self-expression in social interaction, and may predispose to chronic emotional distress. Accordingly, the DS14 comprises a 7-item subscale measuring negative affectivity (tendency to experience negative emotions as the first component of Type D) and a 7-item subscale measuring social inhibition (tendency to inhibit self expression as the second component of Type D). Each item is measured on a 5-point Likert scale ranging from 0 to 4 and each subscale can have a range of scores of between 0 and 28. The Greek version of the DS14 was translated using the procedure recommended by the World Health Organization http://www.who.int/substance_abuse/research_tools/translation/en/index.html.

As this is the first time this scale is used in Greece, in the current paper we also present data on its psychometric properties. The internal consistency of the overall scale was very good with a Cronbach's alpha of 0.86. The Cronbach's alpha for the two subscales was 0.79 and 0.81 for social inhibition and negative affectivity respectively. The item-total correlations had a range between 0.44 - 0.77 for the social inhibition sub-scale and 0.51 - 0.79 for the negative affectivity sub-scale. These figures are very similar with those reported by Denollet in his original validation of the DS14 in a Dutch sample [4]. An exploratory factor analysis of the 14 items was performed using the principal factor method in Stata. As found in the original validation [4] there were two main factors with eigenvalues of 5.36 and 1.54 explaining 49% of the variance. After a promax rotation allowing for oblique factors seven items loaded on the negative affectivity factor with a loading ranging between 0.56 to 0.70 and seven on the social inhibition factor with a loading ranging from 0.51 to 0.75. All items loaded on their corresponding trait factor as found in the original validation in the Dutch sample [4]. In addition all items had a loading on the other factor which was less than 0.33 (corresponding to <10% of overlapping variance) and the minimum difference between the two loadings was at least 0.20. The two factors were moderately correlated with r = 0.34. The full results of the internal consistency analysis and the factor analysis are available from the authors.

Participants were classified as showing a type D personality using the criteria suggested in the original validation study [4]. That is, participants who had a score on both sub-scales above the median were classified as Type D positive. The empirically derived cut-off scores used in the present study were >13 for negative affectivity and >10 for social inhibition.

### Assessment of Anxiety and Depressive symptoms

Anxiety and depressive symptoms were assessed by a validated Greek version of the Hospital Anxiety and Depression Scale (HADS) [15]. The HADS is a brief self-assessment scale assessing the two most common aspects of psychological distress found in hospital settings: anxiety and depression [16]. The scale consists of 14 items, seven for each subscale, and each item is rated on a four-point (0-3) scale. The scoring system ranges from the absence of a symptom or the presence of positive features (scoring 0) to the maximal presentation of symptoms or the absence of positive features (scoring 3). There is no single generally accepted cut-off score for the HADS, especially for the population of the current study. For this reason, in the present study we used the 75^th ^percentile as an arbitrary cut-off to denote clinically significant symptomatology. The 75^th ^percentile was the score of 7 for the depression subscale and the score of 10 for the anxiety subscale and therefore all those scoring above (and not including) this cut-off were considered as "cases" of depression or anxiety respectively.

### Statistical analysis

Preliminary analysis was performed to ensure no violation of the assumptions of linearity and normality. The Shapiro-Wilk test was used to evaluate whether each parameter followed a Gaussian distribution. Data were expressed as mean ± SD, except for non-Gaussian parameters, which were presented as median (range). A comparison of continuous variables was performed by an unpaired two-tailed Student's t-test for normally distributed variables and a Mann-Whitney test for non-normally distributed variables, while chi^2^-tests were used for categorical variables.

The strength of associations of clinical parameters, anxiety and depressive symptoms as well as type D personality with the MetS were assessed by means of logistic regression analysis, comparing persons who met the diagnostic criteria for the MetS and those who did not meet the criteria for the diagnosis of the MetS. These associations were first tested in univariate analysis. Multivariate analysis was then performed by binary logistic regression analysis, which allows adjustment for all potential confounding factors, including demographic, clinical parameters as well as the presence of anxiety or depressive symptoms. Significance levels were set at p < 0.05 in all cases. Stata version 9.0 was used for the analysis (StataCorp, College Station, Texas).

## Results

### Prevalence of type D personality, anxiety and depressive symptoms

Baseline demographic, clinical and laboratory characteristics of the study population are shown in Table 1. Presence of anxiety and depressive symptoms above the cut-off were more common among individuals with MetS as compared to controls (Table 1). The prevalence of type D personality was significantly higher in individuals with the MetS compared with controls (44% versus 15%, in cases with the MetS and controls, respectively, X^2^_(1)_= 33.43, p < 0.001). Each component of type D personality, social inhibition and negative affectivity, was more prevalent in the MetS group than in the control group (Table 1).

**Table 1:** Demographic, clinical/laboratory and psychological features of 359 attenders of an outpatient lipid clinic by metabolic syndrome case status.

	Cases of Metabolic Syndrome (N = 206) Mean (SD/Range) OR N (%)	Control Group (N = 153) Mean (SD/Range) OR N (%)
Age, years	58 (11)	50 (12)**
Male, N (%)	93 (45)	81 (53)
Current smokers, N (%)	43 (21)	39 (26)
BMI, kg/m^2^	30 (6)	26 (4)**
Systolic BP, mm Hg	140 (15)	125 (14)**
Diastolic BP, mm Hg	86 (9)	80 (9)**
Waist circumference, cm	104 (10)	93 (11)**
Fasting blood glucose, mg/dL	107 (17)	93 (11)**
Total cholesterol, mg/dL	235 (51)	207 (40)**
TG, mg/dL	189 (45-934)	122 (34-389)**
HDL-C, mg/dL	49 (13)	52 (14)*
LDL-C, mg/dL	148 (47)	130 (37)**
**Psychological Variables**		
***Type D ***personality, N (%)	90 (44)	23 (15)**
Social inhibition criteria, N (%)	124 (60)	49 (32)**
Negative affectivity criteria, N (%)	123 (60)	41 (27)**
***Depressive symptoms above the cut-off***, N (%)	67 (33)	22 (14)**
***Anxiety symptoms above the cut-off***, N (%)	60 (29)	27 (18)*

-Abbreviations: BMI, body mass index; BP, blood pressure; TG, triglyceride; HDL-C, high-density lipoprotein cholesterol; LDL-C, low-density lipoprotein cholesterol; *p < 0.01 for the comparison between groups, **p < 0.001 for the comparison between groups; Values are expressed as mean (standard deviation) [except TG - median (range)] for continuous variables and number (percentage) for categorical variables.

### Association between type D personality and the metabolic syndrome

In univariate analyses, Type D personality was strongly associated with the presence of MetS (odds ratio: 4.39; 95% confidence interval: 2.60 - 7.39, see Table 2). Both components of this type of personality (i.e. negative affectivity and social inhibition) resulted in significant association with the presence of MetS. Persons with depressive and anxiety symptoms above the cut-off were more likely to be cases of the MetS (Table 2).

**Table 2:** Crude (unadjusted) odds ratios for fulfilling criteria for metabolic syndrome in 359 attenders of an outpatient lipid clinic.

	Odds ratio (95% CI)	p
Components of the Metabolic Syndrome* (European IDF definition)		
High fasting blood glucose (≥100 mg/dl)	11.62 (6.90-19.57)	<0.001
Low HDL-C (<40 mg/dl in ♂ or <50 mg/dl in ♀)	2.44 (1.53-3.91)	<0.001
High TG (≥ 150 mg/dl)	8.93 (5.42-14.72)	<0.001
High BP (≥ 130/85 mmHg)	7.60 (4.62-12.48)	<0.001
**Psychological Variables**		
**Type D personality**	4.39 (2.60-7.39)	<0.001
Negative affectivity sub-component of type D personality	4.05 (2.57-6.37)	<0.001
Social inhibition sub-component of type D personality	3.21 (2.07-4.98)	<0.001
**Depressive symptoms above the cut-off**	2.87 (1.68-4.91)	<0.001
**Anxiety symptoms above the cut-off**	1.92 (1.15-3.20)	0.01

*Please note that increased waist circumference has been omitted because by definition all cases met this criterion. Abbreviations: IDF: International Diabetes Federation; TG: triglyceride; BP: blood pressure; HDL-C: high-density lipoprotein cholesterol; CI, confidence intervals

Results of the multivariate analysis are shown in Table 3. The presence of Type D personality was significantly associated with MetS independently of clinical factors, anxiety and depressive symptoms (odds ratio: 3.47; 95% CI: 1.90-6.33, see Table 3, model 1). We then examined the two sub-components of type D personality (i.e. negative affectivity and social inhibition), which were entered to the model as independent variables instead of type D personality (Table 3, model 2). In this analysis, both sub-components resulted in a significant association with the MetS independently of other clinical and psychological factors (Table 3, model 2). Presence of depressive symptoms above the cut-off also showed evidence of independent statistical association with MetS in the multivariate analysis in contrast to presence of anxiety symptoms above the cut-off which was not significant in the adjusted model. We also tested for an interaction between Type D personality and presence of depressive symptoms above the cut-off but the interaction term was not significant (likelihood ratio test = 0.32 on 1 degree of freedom, p = 0.57).

**Table 3:** Adjusted odds ratios for fulfilling criteria for metabolic syndrome in 359 attenders of an outpatient lipid clinic*.

	Model 1		Model 2	
	Odds ratio (95% CI)	p	Odds ratio (95% CI)	p
**Type D personality**	3.47 (1.90-6.33)	<0.001	NA	NA
Negative affectivity sub-component of type D personality	NA	NA	2.75 (1.56-4.83)	<0.001
Social inhibition sub-component of type D personality	NA	NA	2.03 (1.20-3.45)	0.009
**Depressive symptoms above the cut-off**	2.59 (1.36-4.94)	0.004	2.14 (1.10-4.13)	0.02
**Anxiety symptoms above the cut-off**	0.98 (0.51-1.89)	0.95	0.89 (0.46-1.72)	0.72
**Smoking status**	1.35 (0.72-2.52)	0.34	1.42 (0.75-2.67)	0.28
**Age**	1.04 (1.01-1.07)	0.02	1.04 (1.01-1.07)	0.02

*Variables included in models: Age, gender, marital status, educational status, employment status, household income, smoking status, anxiety symptoms above cut-off, depressive symptoms above cut-off and type D personality (model 1) or sub-components of type D personality [negative affectivity and social inhibition] (model 2).

Abbreviations: CI, confidence interval; NA, not applicable.

### Association between type D personality and the components of the metabolic syndrome

Table 4 shows the association between type D personality and the separate components of the metabolic syndrome. In the adjusted analyses, fasting blood glucose ≥100 mg/dl) and higher TG levels were found to be significantly associated, while higher blood pressure was marginally non-significant.

**Table 4:** Association between Type D personality and the various components of the metabolic syndrome in 359 attenders of an outpatient lipid clinic.

Dependent Variable: Component of the Metabolic Syndrome	Independent Variable: Type D Personality			
**(European IDF definition)**	**Crude Odds ratios** **(95% CI)**	**p**	Adjusted Odds ratios* (95% CI)	**p**

Increased waist circumference (≥94 cm in ♂ or ≥80 cm in ♀)	2.43 (1.18-5.01)	0.02	1.71 (0.73 - 3.99)	0.21

High fasting blood glucose (≥100 mg/dl)	2.53 (1.59 - 4.00)	<0.001	**2.13 (1.23-3.66)**	0.006

Low HDL-C (<40 mg/dl in ♂ or <50 mg/dl in ♀)	1.22 (0.76 - 1.95)	0.41	1.36 (0.78-2.35)	0.27

High TG (≥ 150 mg/dl)	2.19 (1.38-3.45)	0.001	**2.17 (1.28 - 3.68)**	0.004

High BP (≥ 130/85 mmHg)	2.54 (1.50-4.29)	0.001	1.76 (0.97-3.19)	0.06

Odds ratios from separate logistic regression models with each of the components of the metabolic syndrome as the dependent variable and Type D personality as the independent variable. *Odds ratios adjusted for: Age, gender, marital status, educational status, employment status, household income, smoking status, anxiety symptoms above cut-off and depressive symptoms above cut-off. Abbreviations: IDF: International Diabetes Federation; CI: confidence intervals

## Discussion

In a population attending a university-based outpatient lipid clinic, we found that type D personality was significantly associated with a diagnosis of metabolic syndrome. Of interest, this association remained even after adjustment for potential confounding factors, including demographic and clinical parameters as well as the presence of anxiety and depressive symptoms. Multivariate analysis revealed that both components of type D personality resulted in an independent association with the presence of metabolic syndrome. On the other hand, the particular diagnostic components of the metabolic syndrome associated with type D personality were fasting blood glucose ≥100 mg/dl and TG levels ≥ 150 mg/dl..

Our results must be interpreted in the light of certain limitations. Namely, in the context of our cross-sectional study it was not possible to examine the temporal association between type D personality and MetS. Although type D personality is considered to be a stable trait we do not have data on the stability of this construct and the possibility of reverse causality cannot be ruled out. Longitudinal studies should explore further this issue. There is also the possibility of measurement errors, especially in the assessment of psychiatric symptoms. The latter were crudely assessed with a simple self-reported instrument and there is the possibility of misclassification. However, this will be most likely of a random nature and a systematic error is unlikely. Similarly, the assessment of type D personality is based on a simple self-reported instrument. In addition, the type D construct is a relatively recent one and its usefulness and stability over time remain to be established for a range of medical conditions. Finally, in our analyses we did not adjust for lifestyle factors (exercise and diet) and family history of CHD in first degree relatives as these variables were not available.

To date, previous observational studies suggest that type D personality may be related with worse outcomes in patients with established atherosclerosis, including CHD or peripheral artery disease [2,3,17]. Namely, Denollet et al. suggested that patients with CHD who exhibit type D personality are more likely to suffer from a major cardiac event, corresponding to cardiac death, myocardial infarction and cardiac revascularization procedures as compared to those who do not exhibit this type of personality [3]. In addition to that, a recent report suggested that type D personality may serve as an independent predictor of all-cause mortality among patients with peripheral artery disease [17]. Furthermore, it has been shown that this type of personality may be associated with impaired health status and increased mortality rate in patients with chronic heart failure [18,19]. Another example of this kind of psychosomatic association is that anxious patients with implantable cardiac defibrillator who exhibit type D personality are more susceptible to suffer ventricular arrhythmias [20].

Another important clinical and research point is to identify mechanisms to explain this potential association between type D personality and the pathogenesis or the progression of atherosclerotic vascular disease. Several studies demonstrated a link between type D personality and biomarkers which may increase cardiovascular risk. For instance, persons with type D personality may exhibit higher circulating levels of several pro-inflammatory cytokines, including tumor necrosis factor-a (TNA-a) and its soluble receptors 1 and 2. These cytokines are known to be contributors in the pathogenesis of CHD as well as predictors of mortality among patients with CHD [6]. Furthermore, an elevated cortisol secretion in response to stress as well as higher cortisol output, as a result of a prolonged disruption of adrenal-pituitary axis function, was noted among persons with type D personality [21,22]. Morning cortisol secretion was noted to positively correlate with significant coronary artery stenosis in women having survived an acute coronary syndrome [23]. It is important to appreciate that type D personality may increase cardiovascular risk by favoring behavioral patterns including an unhealthy lifestyle as well as poor compliance to treatment [24].

To date, there are no sufficient data to establish an association between type D personality and 'traditional' cardiovascular risk factors. In this study, a strong and independent association was demonstrated between this type of personality and MetS. MetS is an insulin resistant state which comprises a clustering of traditional cardiovascular risk factors, including elevated blood pressure and TG levels, low concentration of HDL-cholesterol as well as impaired fasting glucose [10]. Therefore, MetS should be considered an early stage in the pathway of atherosclerotic vascular disease progression [10]. Our results may provide an additional mechanism to explain adverse cardiovascular outcomes among persons who exhibit this type of personality.

Since the concept of type D personality is relatively new, few assumptions could be made to explain a potential association between this type of personality and MetS. As described previously, type D personality has been associated with a prolonged disruption of adrenal-pituitary axis function [23]. Hypothalamus-pituitary-adrenal axis dysregulation, resulting in elevated cortisol levels, has been associated with hypertension, hyperlipidemia and insulin resistance, which are the main components of MetS [25]. Furthermore, there is tendency of persons with type D personality to experience anxiety or depressive symptoms which are both associated with MetS [26-28]. The high prevalence of these psychiatric disorders in individuals with type D personality, also observed in our study, may be of help to explain the association between type D personality and the MetS. This may hold true considering that negative health behaviors as well as hypothalamic and sympathetic dysregulation related to these psychological disorders may increase the risk for the development of the MetS, particularly for its components of central adiposity and insulin resistance [26]. Consistently, according to our univariate analysis, type D personality was significantly associated with fasting blood glucose and waist circumference measurements (although the latter was reduced after adjustment, see Table 4). Therefore, it could be hypothesized that the association between type D personality and MetS may be mediated through the enhanced prevalence of anxiety or depressive symptoms in type D personality (also evident in our study). However, in our multivariate analysis, type D personality was found to be the most significant predictor of MetS independently of the presence of either anxiety or depressive symptoms. It should be noted however, that the cross-sectional nature of our study is limiting our ability to understand the complex relationship between anxiety, depression, type D personality and their impact on the development of the MetS.

### Future perspective

In this cross-sectional study we identified a type of personality, characterized by increased emotional distress and avoidance of social contacts, as a strong and independent predictor of the MetS. Since the MetS serves as a prodromal stage in the development of atherosclerotic vascular disease, it would be interesting to study whether modification of these personality characteristics could reduce the risk for the progression of atherosclerotic vascular disease giving an opportunity for primary prevention. Future studies should aim to explore this possibility.

## Competing interests

The authors declare that they have no competing interests.

## Authors' contributions

DT and PS were responsible for the conception of the study. DT organized and carried out the data collection and helped in the drafting of the manuscript. MSK helped in the drafting of the manuscript and in the statistical analysis. PS supervised the project, designed the methodology, carried out the main statistical analysis, helped in the drafting of the manuscript and interpretation of the results and revised the manuscript according to the comments received by the peer review process. HJM, MSE and VM made critical comments that helped in the interpretation of the results. HJM and MSE additionally helped in the recruitment of the patients. VM additionally helped in the general management and supervision of the project. All authors read and approved the final manuscript.
